# The Ankyrin Repeat Domain 49 (ANKRD49) Augments Autophagy of Serum-Starved GC-1 Cells through the NF-κB Pathway

**DOI:** 10.1371/journal.pone.0128551

**Published:** 2015-06-04

**Authors:** Hai-long Wang, Sha-sha Fan, Min Pang, Yi-heng Liu, Min Guo, Jun-bo Liang, Jian-lin Zhang, Bao-feng Yu, Rui Guo, Jun Xie, Guo-ping Zheng

**Affiliations:** 1 Department of Biochemistry and Molecular Biology, Shanxi Medical University, Taiyuan, Shanxi, 030001, PR China; 2 Department of Respiratory, the First Affiliated Hospital, Shanxi Medical University, Taiyuan, Shanxi, 030001, PR China; 3 Class 041002, Department of Anestesioloy, Shanxi Medical University, Taiyuan, Shanxi, 030001, PR China; 4 Center of Laboratory Animal, Shanxi Medical University, Taiyuan, Shanxi, 030001, PR China; 5 State Key Laboratory of Medical Molecular Biology, Institute of Basic Medical Sciences, Chinese Academy of Medical Sciences, Peking Union Medical College, Tsinghua University, 5 Dong Dan San Tiao, Beijing, 100005, China; Cardiff University, UNITED KINGDOM

## Abstract

The ankyrin repeat domain 49 (ANKRD49) is an evolutionarily conserved protein highly expressed in testes. However, the function of ANKRD49 in spermatogenesis is unknown. In this study, we found that ANKRD49 resides primarily in nucleus of spermatogonia, spermatocytes and round spermatids. ANKRD49 overexpression augments starvation-induced autophagy in male germ GC-1 cells whereas shRNA knockdown of ANKRD49 attenuates the autophagy. Inhibition of NF-κB pathway by its inhibitors or p65 siRNA prevents the ANKRD49-dependent autophagy augmentation, demonstrating that ANKRD49 enhances autophagy via NF-κB pathway. Our findings suggest that ANKRD49 plays an important role in spermatogenesis via promotion of autophagy-dependent survival.

## Introduction

Mammalian spermatogenesis is a highly ordered process that includes mitosis of spermatogonial stem cells, meiosis of spermatocytes and spermiogenesis [1]. This process depends on balance of germ cell proliferation, differentiation and death in the testes [2]. During spermatogenic differentiation, over half of the differentiating spermatogenic cells die before they mature into spermatozoa. Although apoptosis is the main cause of cell death in spermatogenesis [3], it is not the only way of genetically programmed death. Autophagy is referred as type II programmed cell death for lacking caspase activation or DNA fragmentation, the two classical characteristics of apoptosis [4]. However, autophagy may also promote cell survival under oxidative stress, virus infection and nutrient deprivation [5–7]. Previous studies have shown that multiple genes regulating autophagy are involved in spermatogenesis, including Atg7 and the gene encoding the GAGA protein [8, 9]. They are found to be cytoprotective and essential for germ cell maturation. The ankyrin repeat domain 49 (ANKRD49) contains four ankyrin repeats, a motif of 30 to 34 amino acid residues [10] that was first identified in the yeast sequences Swi6p, Cdc10p and Notch [11]. Families of ankyrin repeat proteins that mediate protein-protein interactions have been associated with cancer progression [12]. It has been reported that ANKRD49 is highly expressed in low invasive lung cancer cell lines [13] and is expressed at low levels in rat lateral habenula in a depression model of escitalopram responders [14]. However, the function of ANKRD49 is unknown.

In present study, we found that ANKRD49 is highly expressed in mouse testes and located predominantly in nucleus. Importantly, biological function of ANKRD49 in modulating of autophagy via NF-κB pathway has been investigated. Our results revealed novel insight into biological function and molecular mechanisms of ANKRD49 in spermatogenesis.

## Materials and Methods

### Tissue and cells

BALB/c mice were purchased from Laboratory Animal Center of Shanxi Medical University. Testes tissue obtained from male BALB/c mice at different ages (from one to eight weeks) were used to examine the temporal and spatial expression patterns of ANKRD49 in male germ cells. Mice were anaesthetized with sodium pentobarbital (1.5%,20 ml/body weight) for tissue collection and euthanasia after tissue collection. Animal carcases were stored on site in a -20°C freezer and later processed through a roto-autoclave and then into deep land fill by Laboratory Animal Center of Shanxi Medical University. All experimental and surgical procedures were reviewed and approved by the Ethics Committee of Animal Experiments of Shanxi Medical University.

GC-1 spg and GC-2spd cells were purchased from American Type Culture Collection (ATCC, USA) and cultured in DMEM (HyClone, USA) with 10% foetal bovine serum (FBS, HyClone, USA). TM-3 and TM-4 cells were obtained from the Cell Culture Center of the Chinese Academy of Medical Sciences (Beijing, China). TM3 cells were maintained in RPMI 1640 medium (HyClone, USA) with 10% FBS. TM4 cells were grown in a mixture of DMEM and Ham's F12 medium (HyClone, USA) plus 5% horse serum (Sigma, USA) and 5% FBS in 5% CO_2_ at 37°C.

### Expression plasmids, transfection and construction of GC-1 stable cells

GFP-LC3 plasmid DNA was purchased from Shanghai GenePharma Co., Ltd (Shanhai, China). NF-kB–driven luciferase reporter and Renilla luciferase construct were purchased from Beyotime Institute of Biotechnology, China. Mouse NF-κB p65 siRNA (sc-44213, sc-29411) and control siRNA (sc-37007) were purchased from Santa Cruz (USA). For construction of mouse *ANKRD49* expression plasmids using pMSCVpuro (Clontech, USA), the coding region of mouse *ANKRD49* (accession number: NM_019683.3, http://www.ncbi.nlm.nih.gov/nuccore/ NM_019683.3) was amplified by PCR from a mouse testes cDNA library. The forward primer is 5’-ggaAGATCTGCCACCatggaaaaagaaaaaggaaat gatg-3’. The reverse primer is 5’-ccgctcgagTTACTTGTCATCGTCGTCCT TGTAGTCAGACTGAGGTGAAGAATTTGTAC-3’, including a Flag-tag sequence (underlined). PCR products were cloned into the pMSCVpuro vector at the *Bgl* II / *Xho* I (Takara, Japan) sites.

The pRNAT-H1.1/Hygro plasmid (GenScript, USA) was used to express ANKRD49 small-hairpin RNA (shRNA) and control shRNA in GC-1 cells. Briefly, chemically synthesized oligonucleotides were annealed and inserted into the pRNATH1.1/Hygro vector between the *Bam*H I and *Hind* III (Takara, Japan) sites. All constructs were confirmed by sequencing. Two targeted mouse ANKRD49 sequences are, 5’-GATCCAAGCAAATTGCTTC-3’ (1#) and 5’-ATTGCGGAA GGCTGTACAA-3’ (2#). The negative control sequence is 5’-TAAGGCTATGAA GAGATAC-3’.

Lipofectamine 2000 (Invitrogen, USA) was used for plasmid transfections. To generate stable GC-1/Con and GC-1/ANKRD49-Flag cell lines, the pMSCVpuro-ANKRD9-Flag plasmid was transfected into GC-1 cells and selected by puromycin (2 μg/ml) (Sigma, USA) [15]. GC-1 cell lines were generated that stably express pRNAT-H1.1/Hygro-negative, pRNAT-H1.1/Hygro-1# and pRNAT-H1.1/Hygro-2# [16].

### Antibodies

The ANKRD49 rabbit polyclonal antibody was purchased from Abcam (U.K). The Flag mouse monoclonal, Beclin 1, LC3A/B, p65, p62 and GAPDH antibodies were purchased from Cell Signaling Technology (USA); the β-actin and cIAP2 antibodies were purchased from Santa Cruz Biotechnology (USA). HRP-conjugated secondary antibodies were obtained from Zhongshanjinqiao Company (China). Alexa Fluor 488-conjugated goat anti-rabbit antibody, Alexa Fluor 488 goat anti-mouse antibody and Alexa Fluor 546-conjugated goat anti-rabbit antibody were purchased from Life Technologies (USA).

### Cell treatment and Western blot

GC-1 cells stably expressing either ANKRD49 or ANKRD49 shRNA at 80% confluence were treated with serum-free media for 24 h as starvation treatment; the NF-κB pathway inhibitors, pyrrolidine dithiocarbamate (PDTC) (50 μM) (Sigma, USA) or BAY 11–7082 (10 μM) (Santa Cruz, USA) was added to complete media for 2 h prior to the serum-free media treatment. Nuclear proteins were obtained using NE-PER Nuclear and Cytoplasmic Extraction Reagents (Pierce, USA), total proteins were extracted with RIPA buffer and quantified using the BCA protein assay reagent (Thermo Scientific, USA). Protein samples were separated by 12% SDS-PAGE and transferred to a 0.2 μm PVDF membrane (Millipore, USA). Membranes were blocked in 5% skim milk for 1 h at room temperature, followed by an overnight incubation at 4°C with primary antibody followed by incubation with their corresponding HRP-IgGs, then visualized using an ECL blot detection system (Transgene, Beijing, China). Band intensities were quantified using a Tanon 1600 Gel Image Analysis System.

### Quantitative real-time-PCR (qRT-PCR)

Total RNA from mouse tissues and GC-1 cells was extracted with TRIzol Reagent (CWBIO, Beijing, China), and RT-PCR was performed using Applied Biosystems real-time PCR instruments (USA) and UltraSYBR Two Step RT-qPCR Kit (CWBIO, Beijing, China) according to the manufacturer’s instructions. The 2^−ΔΔCT^ method was used to calculate the relative levels of ANKRD49 mRNA normalized against housekeeping gene GAPDH. The primers for qRT-PCR were as follows: forward primer 5’-ACACCTGATTCCCACTGG-3’ and reverse primer 5’-GCACTGTAGC AAGCCGAT-3’ were used to amplify ANKRD49, and forward primer 5’-TGAGTACGTCGTGGAGTCCA-3’ and reverse primer 5’-TAGACTCCACGACATACTCA-3’ were used to amplify GAPDH.

### Immunohistochemistry (IHC) and immunofluorescence (IF)

Mouse testes tissues were fixed with 4% paraformaldehyde, sliced into transverse sections and embedded in paraffin. 4 μm sections were used for IHC staining, and analysed as previously described [17]. Subconfluent cells grown on glass coverslips with different treatment were fixed with 4% paraformaldehyde in PBS for 20 min at RT, blocked with 3% bovine serum albumin (BSA, Sigma, USA) in PBS, and incubated overnight at 4°C with a rabbit anti-Beclin 1 antibody (1:100) or a rabbit anti-LC 3 antibody (1:100). Cells were then stained with Alexa Fluor 488-conjugated goat anti-rabbit antibody (1:200), Alexa Fluor 546-conjugated goat anti-rabbit antibody (1:400) and counterstained with DAPI (Sigma, USA) in PBS for 1 h at RT. Fluorescence images were analyzed using a FV1000 Confocal Laser Scanning Microscopy (Olympus, Japan).

### Transmission electron microscopy (TEM) and fluorescence microscopy

GC-1/Con and GC-1/ANKRD49-Flag cells were cultured with serum-free media for 24 h. Standard TEM was performed as previously described [18]. For fluorescence microscopy, GC-1/Con and GC-1/ANKRD49-Flag cells were transfected with a plasmid expressing the green fluorescent protein (GFP)-LC3. The transfected cells were treated with or without PDTC or BAY 11–7082 in serum-free media for 24 h. GFP-LC3 was detected by the FV1000 Confocal Laser Scanning Microscopy.

### NF-κB luciferase reporter assay

GC-1/Con and GC-1/ANKRD49-Flag cells seeded at 1×10^5^ cells/well in 24 well plates were cultured overnight and co-transfected with 0.5 μg NF-κB-driven luciferase reporter and 0.02 μg Renilla luciferase constructs using Lipofectamine 2000 according to the manufacturer’s instructions. 24 h after transfection, the cells were treated with or without PDTC or BAY 11–7082 in serum-free medium for another 24 h. Reporter activities (Firefly and Renilla luciferases) were determined using Dual-Luciferase Reporter Assay System (Promega, USA) by a luminometer (TD-20/20, Turner BioSystems, USA) according to the manufacturer’s instructions. The NF-κB transcriptional activities were expressed as relative luciferase activity calculated by the ratio of Firefly luciferase activity against Renilla luciferase activity.

### Statistical analysis

Data were analysed using Student’s t-test and presented as the mean ± SD. The results are considered statistically significant when *p*<0.05.

## Results

### ANKRD49 is highly expressed in testes and is likely to be involved in spermatogenesis

**Fig 1:**
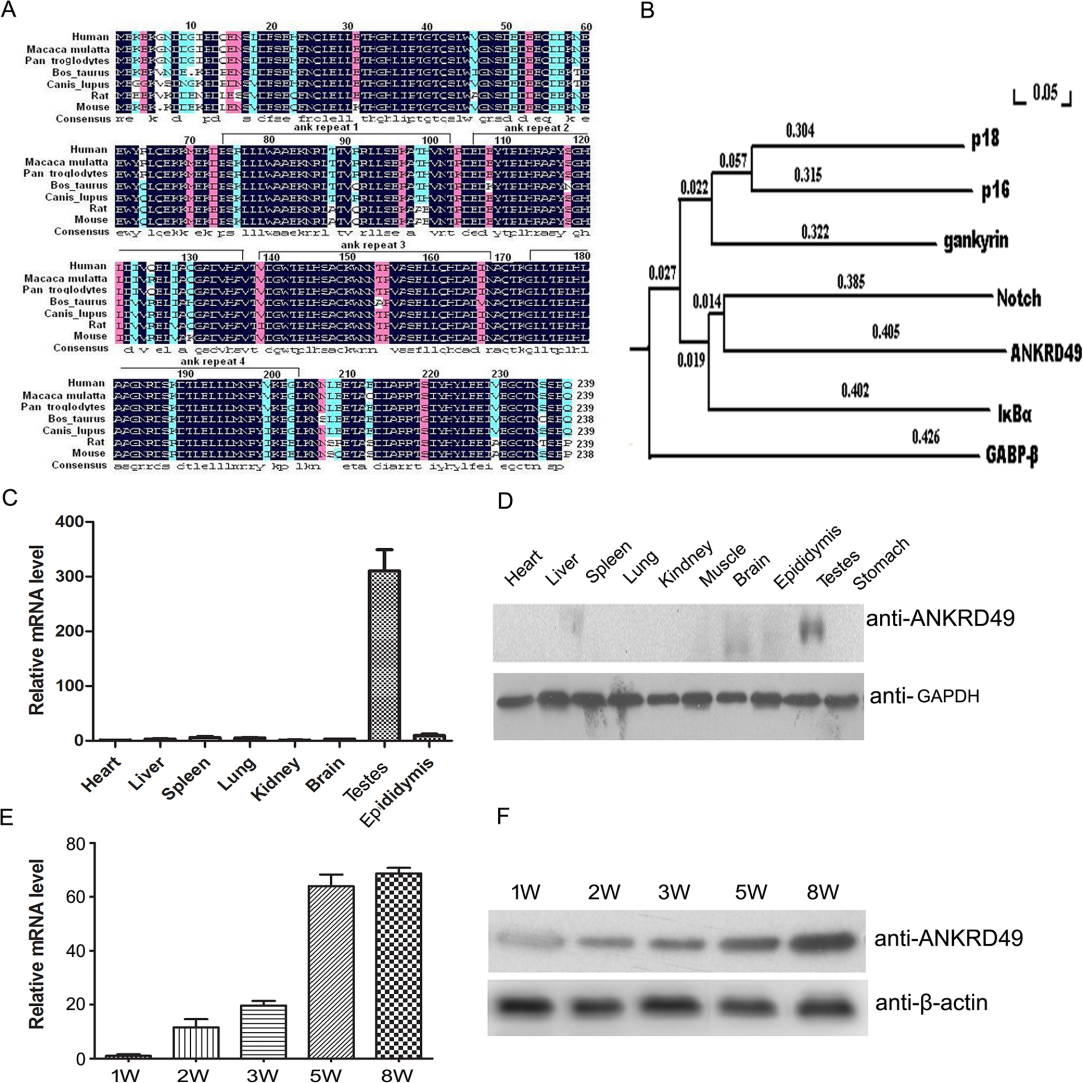
The expression of ANKRD49 in a panel of mouse tissues and its temporal expression in developing mouse testes. (A) Alignment of the amino acid sequence of mouse ANKRD49 (AAH19777.1) and human (NP_060174.2), Macaca mulatta (AFH27401.1), Pan troglodytes (JAA43031.1), Bos_aurus (NP_001014965.1), Canis_lupus (XP_005633413.1) and rat (AAI61982.1). The alignment was performed by DNAMAN (Lynnon, Quebec, Canada). Homology levels are highlighted in different colours. Black: 100%; Pink: 75%; Blue: 50%. (B) Phylogenetic tree analysis of ANKRD49 and ankyrin repeat family proteins. Numbers indicate branch length. (C) The tissue distribution of ANKRD49 mRNA in adult mice was analysed by quantitative RT-PCR. (D) The expression pattern of ANKRD49 in a panel of tissues from adult mice was determined by Western blot. (E and F) The mRNA and protein levels of ANKRD49 in the testes of mice one to eight weeks old were detected by qRT-PCR and Western blot. GAPDH served as a loading control. Each assay was repeated three times with similar results. W is the abbreviation for ‘‘week.” Each number represents the age of the mice.

Mouse ANKRD49 (gene accession number: NM_019683.3) is located on chromosome 9. It has one transcript of 1753 bps. This gene encodes a 238 amino acid protein which contains four ankyrin repeat domains. As shown in Fig 1A, there are evolutionarily conserved variations in the ANKRD49 protein among different genera, with 93.96% similarity in an amino acid sequence. Further phylogenetic tree analysis shows that ANKRD49 has a branch length similar to other ankyrin repeat domain proteins (Fig 1B). To elucidate the expression patterns of ANKRD49, we isolated mouse tissues and examined ANKRD49 expression using qRT-PCR and Western blot. These experiments show that adult mouse testes exhibit a higher expression of ANKRD49 compared with other tissues examined (Fig 1C and 1D). We also examined testes from mouse of different ages to assess the temporal expression of ANKRD49. As shown in Fig 1E and 1F, ANKRD49 expression exhibits dynamic variation throughout the establishment of spermatogenesis. The lowest level of ANKRD49 mRNA expression appears during the first week after birth. Its mRNA levels increase with age and plateau at eight weeks. ANKRD49 protein levels show similar expression patterns. The temporal special patterns of ANKRD49 expression strongly suggest that ANKRD49 is involved in spermatogenesis.

### The localization of ANKRD49 exhibits a prophase of spermatogenesis

**Fig 2:**
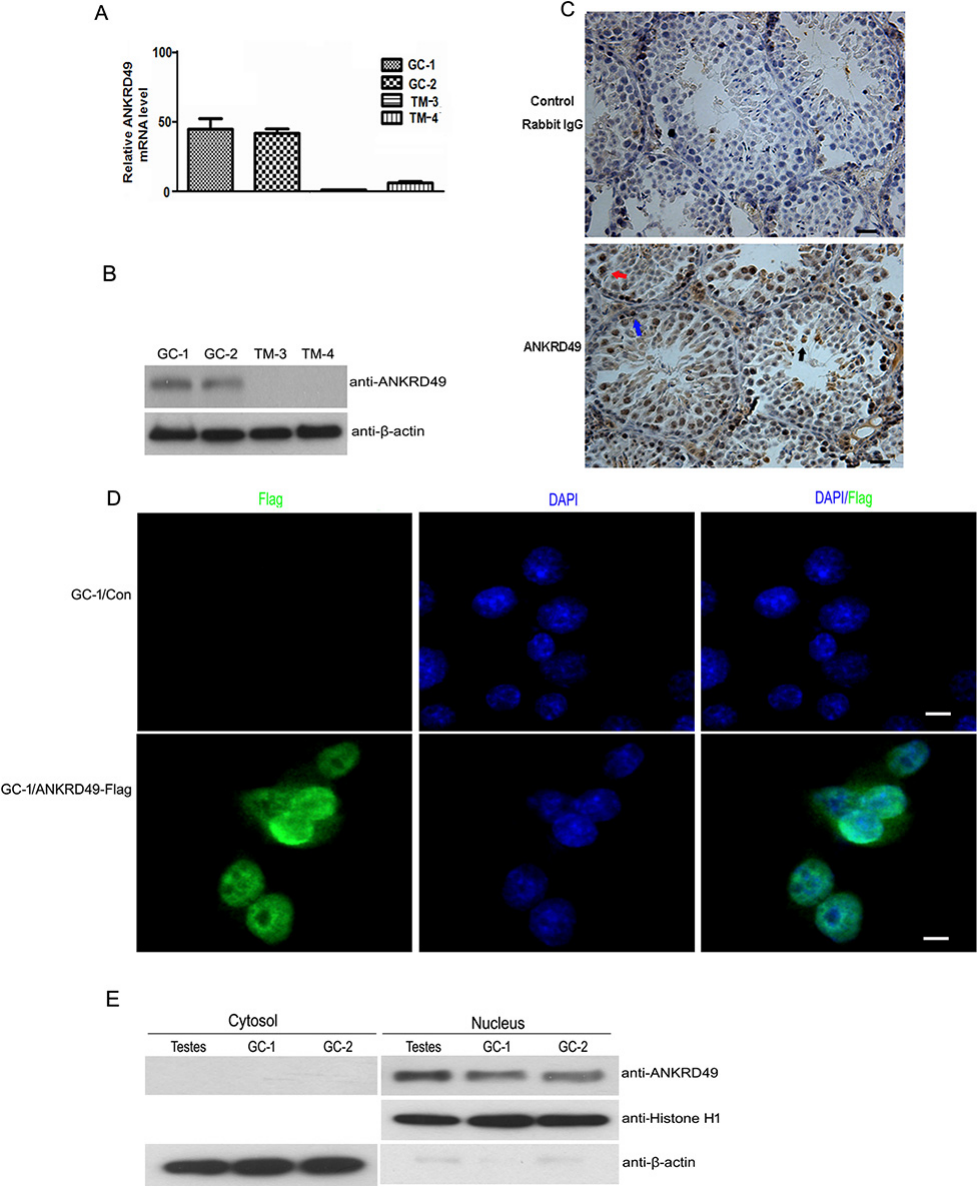
ANKRD49 is detected primarily in spermatogonia, spermatocytes and round spermatids and localizes in the nucleus. (A and B) The cell distribution of ANKRD49 mRNA and protein in four mouse testes-related cell lines were analysed by quantitative RT-PCR (A) and western blot (B). β-actin served as a loading control. (C) IHC analysis was performed with an anti-ANKRD49 polyclonal antibody (bottom panel) to detect the localization of ANKRD49 in adult mouse testes. A negative control was performed using rabbit IgG (upper panel). Blue arrow: spermatogonia; red arrow: spermatocytes; black arrow: round spermatids. Scale bars represent 50 μm. (D) ANKRD49 localizes to the nucleus. GC-1 cells which stably express ANKRD49 were subjected to IF analysis with an anti-Flag antibody followed by incubation with Alexa Fluor 488 goat anti-mouse antibody. Fluorescence signals were analysed using Confocal Laser Scanning Microscopy. Nuclei were stained with DAPI. Scale bars represent 10 μm. (E) The cytoplasm and nuclear proteins were extracted and Western blot assay was performed to detect the distribution of ANKRD49 in adult mouse testes, GC-1 and GC-2 cells. β-actin and histone H1 served as loading controls of cytoplasm and nuclear proteins, respectively. Each assay was repeated three times with similar results.

We further investigated the distribution of ANKRD49 in adult mouse testes to understand the role of ANKRD49 in spermatogenesis. Firstly, we detected the expression patterns of ANKRD49 in GC-1 cells (a mouse-derived spermatogonia cell line), GC-2 cells (a mouse-derived spermatocyte cell line), TM-3 cells (a mouse Leydig cell line) and TM-4 cells (a mouse Sertoli cell line) using qRT-PCR and Western blot. The results show that ANKRD49 is expressed in GC-1 and GC-2 cells (Fig 2A and 2B). Next, we determined the distribution of ANKRD49 in mouse testes using immunohistochemistry assay. Fig 2C shows that ANKRD49 localizes primarily in the spermatogonia, spermatocytes and round spermatids. Moreover, ANKRD49 is found in the nucleus. We then localized ANKRD49 using immunofluorescence assay by an anti-Flag antibody in GC-1 cells that stably express ANKRD49. The immunofluorescence assay also shows that ANKRD49 staining is distributed in the nucleus (Fig 2D). Moreover, we prepared the cytoplasm and nuclear proteins from mouse testis tissue, GC-1 cells and GC-2 cells. Western blot shows that ANKRD49 is detecteded in nucleus fraction (Fig 2E).

### ANKRD49 augments autophagy induced by serum starvation of GC-1 cells derived from the male germ line

**Fig 3:**
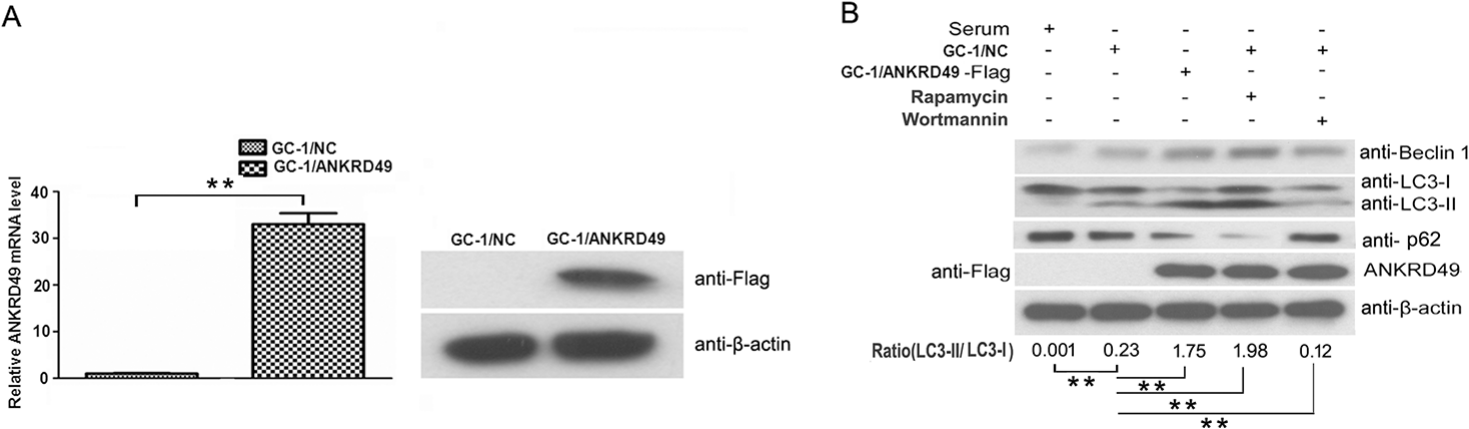
ANKRD49 induces autophagy in serum-starved GC-1 cells. (A) The expression level of ANKRD49 in GC-1 stable cells. Left: mRNA levels, right: protein levels. (B) ANKRD49-induced autophagy, detected by the presence of Beclin 1, LC3-I to LC3-II conversion and p62 were analysed by Western blot; Rapamycin served as an autophagy-positive control and Wortmannin served as an autophagy-negative control, β-actin served as a loading control. One representative of three independent experiments is shown. The quantitative results are presented as the ratio of LC3-II to LC3-I (n = 3). **p < 0.01 indicates significant difference between groups as shown.

**Fig 4:**
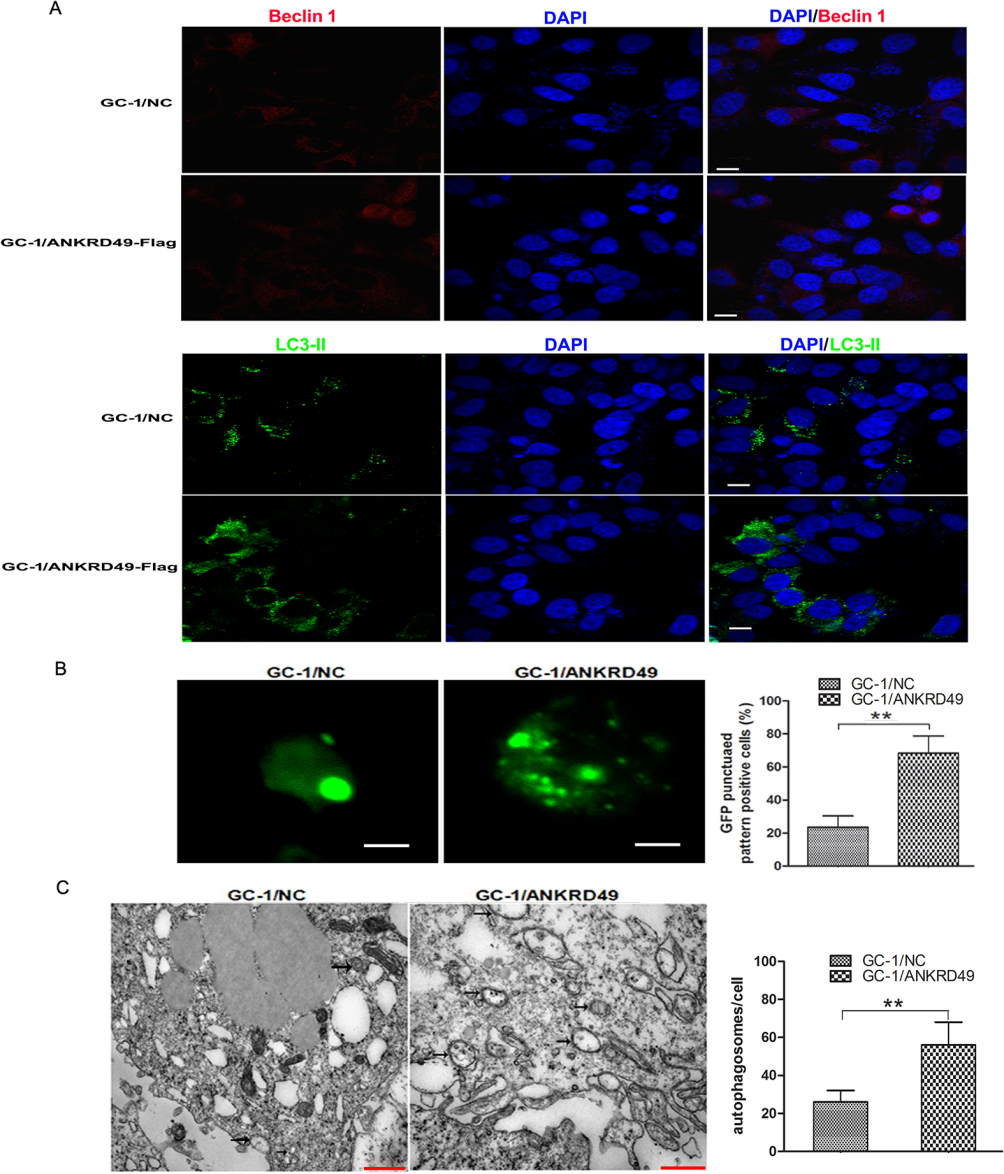
ANKRD49 induces autophagy in serum-starved GC-1 cells. (A) GC-1/ANKRD49-Flag cells and control cells were cultured on glass coverslips and serum-starved for 24 h, labelled with rabbit anti-Beclin 1 (red) and rabbit anti-LC3-II antibody (green) and exposed to DAPI for nuclei visualization (blue). Fluorescence signals were analyzed by using a Confocal Laser Scanning Microscopy Scale bar represents 10 μm. (B) GC-1/ANKRD49-Flag cells and control cells were transfected with GFP-LC3, followed by serum starvation. Punctated GFP-LC3-Ⅱ was observed by a Confocal Laser Scanning Microscopy (left) and a punctuated pattern was shown (right), indicating appearance of autophagy. Scale bar represents 100 μm. **p < 0.01 indicates significant difference between groups as shown. (C) Transmission electron microscopy analysis of serum-starved GC-1/ANKRD49-Flag cells and control cells. GC-1/ANKRD49-Flag cells display more autophagosomes (indicated by black arrows) than those displayed by controls (left). The relative numbers of autophagosomes in different groups are shown (right). Data are presented as the mean ± SD (n = 3) of three independent experiments. **p < 0.01 indicates significant difference between groups as shown. Scale bars represent 2 μm.

To evaluate the putative roles of ANKRD49 in spermatogenesis, we used the GC-1 cell line as a cell model. We have constructed a pMSCVpuro-ANKRD49-Flag eukaryotic expression plasmid and transfected it into GC-1 cells. Stable GC-1/Con and GC-1/ANKRD49-Flag expression cell lines were selected by puromycin, confirmed by qRT-PCR and Western blot (Fig 3A). Given that autophagy has both cytoprotective and apoptotic (or cell death) roles, and that apoptosis is important in spermatogenesis [8, 9],we then examined the occurrence of autophagy in GC-1/Con and GC-1/ANKRD49-Flag cells after serum starvation for 24 h. Beclin 1, LC-3 and p62, the commonly used autophagy markers [19–21], were determined by Western blot and immunofluorescence staining. As illustrated in Fig 3B and Fig 4A, the levels of Beclin 1 and LC3-II in serum-starved GC-1/ANKRD49-Flag cells are greater than those in GC-1/Con cells while the p62 has a counter trend. In addition, the expression of Beclin 1 and LC3-II detected by immunofluorescence, are in accordance with the Western blot assay (Fig 4A). Furthermore, we detected GFP-LC3 dots which are regarded as autophagosomes [22]. GC-1/ANKRD49-Flag and parental cells transfected with GFP-LC3 were treated by serum starvation. As shown in Fig 4B, marked punctate accumulation of GFP-LC3 is observed in GC-1/ANKRD49-Flag cells, demonstrating a high level of autophagy. In addition, TEM-based ultrastructural analysis confirmed the formation of double-membrane vesicles (autophagosomes) (Fig 4C).

**Fig 5:**
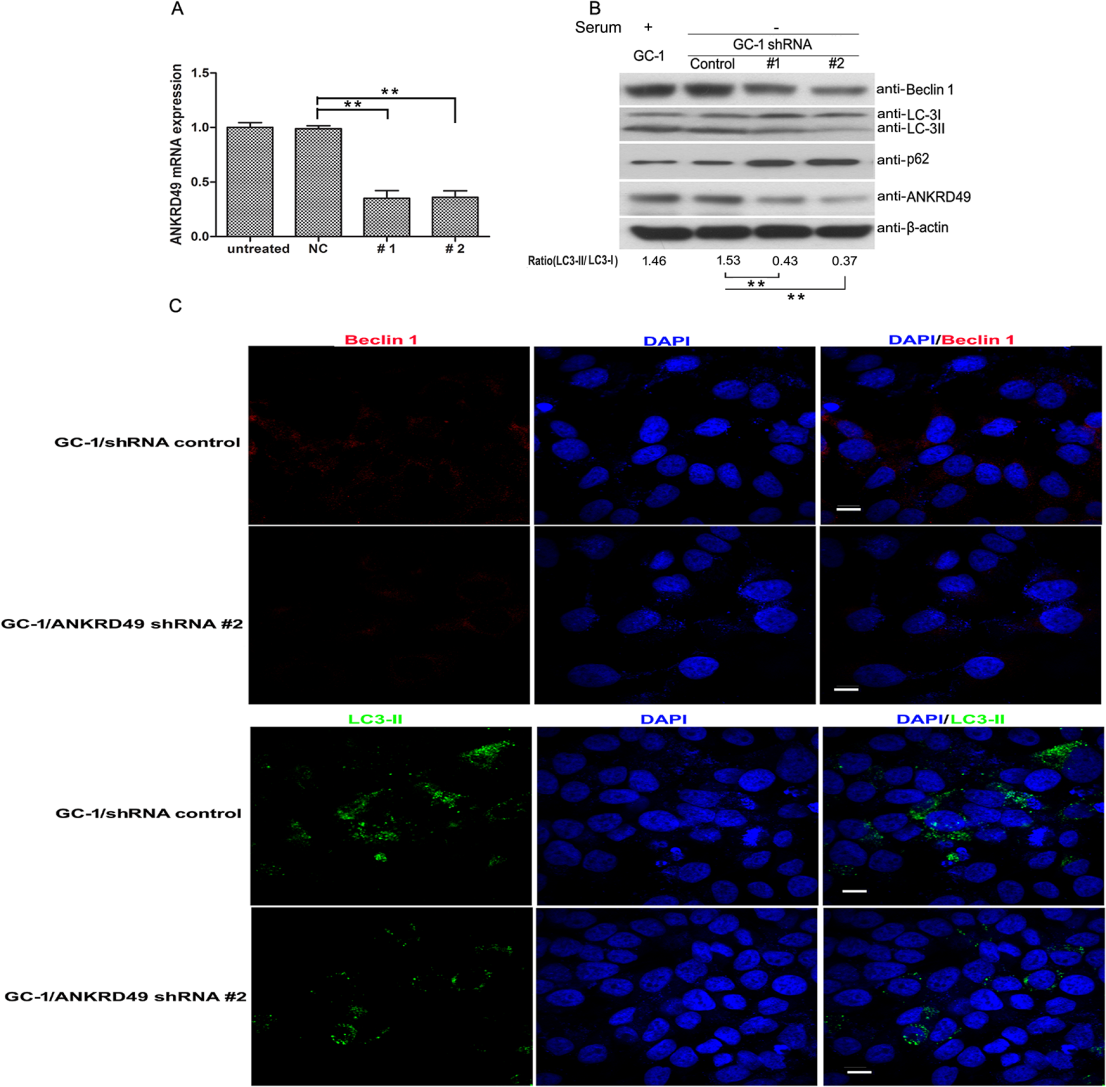
ANKRD49 knockdown decreases autophagy in serum-starved GC-1 cells. (A) GC-1 cells were transfected with RNAi plasmids 1# and 2# against ANKRD49 and a scramble sequence as a negative control. Stable ANKRD49 knockdown GC-1 cell clones were screened by puromycin and identified by qRT-PCR. (B) Western blot analysis of Beclin 1, LC3-Ⅱ, p62 and ANKRD49 in 1#, 2# and negative control GC-1 cells which were serum-starved for 24 h. Control cells were subcultured from a stable GC-1 cell clone expressing a negative control shRNA. β-actin served as a loading control. One representative of three independent experiments is shown. The quantitative results are presented as the ratio of LC3-II to LC3-I (n = 3). **p < 0.01 indicates significant difference between groups as shown. (C) GC-1/ANKRD49-shRNA cells and control cells were cultured on glass coverslips and serum-starved for 24 h, labelled with rabbit anti-Beclin 1 (red) and rabbit anti-LC3-II antibody (green) and exposed to DAPI for nuclei visualization (blue). Fluorescence signals were analyzed by using a Confocal Laser Scanning Microscopy Scale bar represents 10 μm.

To further explore the pro-autophagy function of ANKRD49 in GC-1 cells, stable ANKRD49 knockdown shRNA and negative control shRNA transfected GC-1 cells were generated (Fig 5A). The occurrence of autophagy in ANKRD49 knockdown GC-1 cells were determined after serum starvation. As shown in Fig 5B, the autophagy markers (Beclin 1 and LC3-Ⅱ) are significantly lower in the GC-1 cells with the ANKRD49 knockdown compared with those of negative control while the p62 has a reverse expression pattern. These results demonstrated that ANKRD49 promotes autophagosome formation in GC-1 cells. Moreover, the levels of Beclin 1 and LC3-II were detected by immunofluorescence, and the results are in line with the Western blot assay (Fig 5C).

### ANKRD49-regulated autophagy of GC-1 cells is dependent on the NF-κB pathway

**Fig 6:**
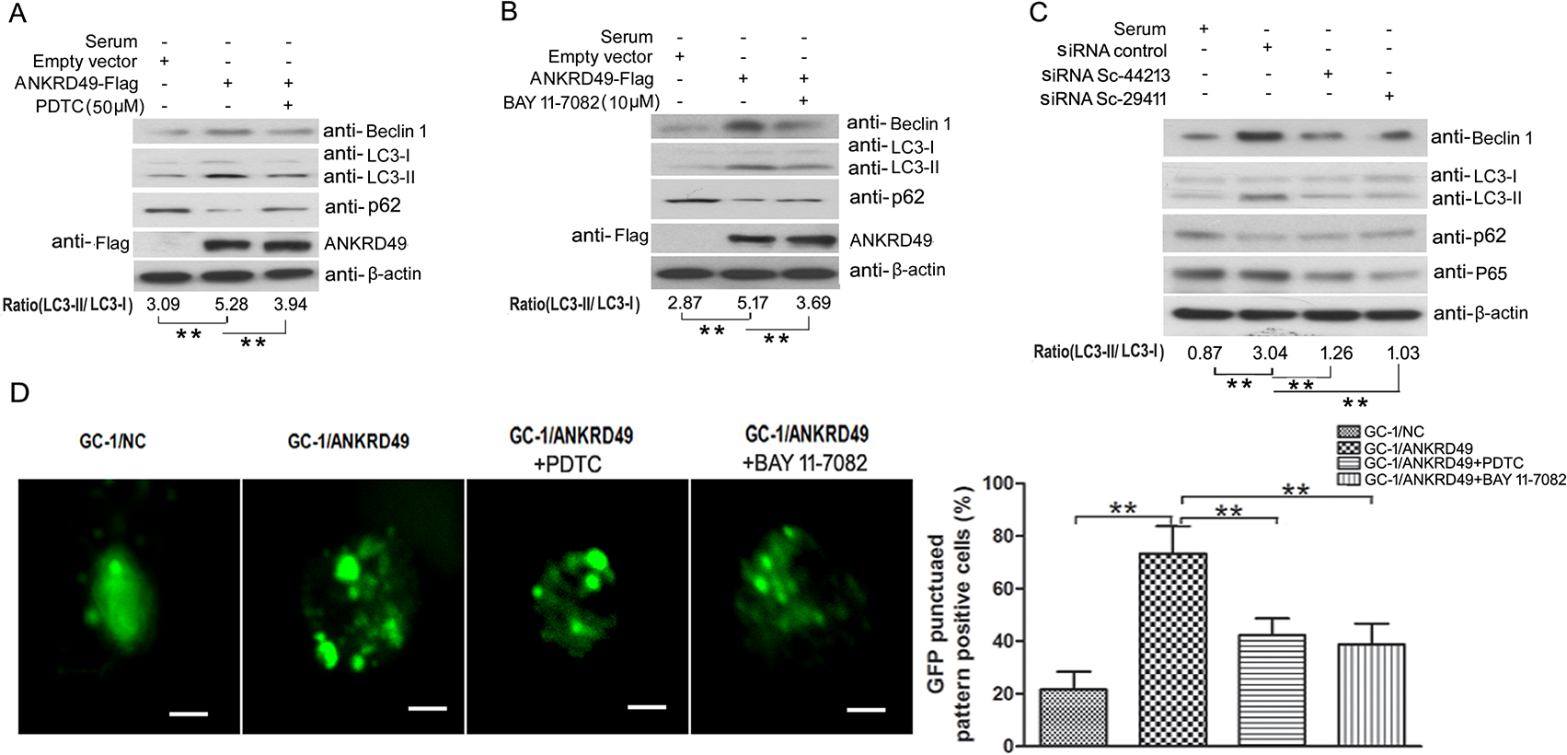
ANKRD49-regulated autophagy induced by serum-starved GC-1 cells is dependent on the NF-κB pathway. (A, B) GC-1/ANKRD49-Flag stable cells were pre-treated with 50 μm PDTC or 10 μm BAY 11–7082 for 2 h prior to treatment with serum-free media for another 24 h, and the cell lysates were prepared and blotted with the indicated antibodies. (C) GC-1/ANKRD49-Flag stable cells were transfected with mouse NF-κB/p65 siRNA and a negative control for 24 h and incubated in serum-free media for another 24 h. Cell lysates were prepared and blotted with the indicated antibodies. β-actin served as a loading control. One representative of three independent experiments is shown. The quantitative results are presented as the ratio of LC3-II to LC3-I (n = 3). **p < 0.01 indicates significant difference between groups as shown. (D) GC-1/ANKRD49-Flag cells and control cells were transfected with GFP-LC3 for 24 h, followed by serum starvation culture with or without PDTC and BAY 11–7082 for another 24 h. Punctated GFP-LC3-Ⅱ was observed by a Confocal Laser Scanning Microscopy (left) and a punctuated pattern was shown (right), indicating appearance of autophagy. Scale bar represents 100 μm. **p < 0.01 indicates significant difference between groups as shown.

It has been reported that NF-κB is involved in the process of autophagy [23, 24]. To investigate whether NF-κB participates in the process of ANKRD49-induced autophagy in GC-1 cells, we examined the ANKRD49-induced autophagy in the presence of NF-κB pathway inhibitors, PDTC and BAY 11–7082 [25, 26]. Fig 6A and 6B shows that in the presence of PDTC or BAY 11–7082, the levels of Beclin 1 and LC3-Ⅱ are decreased in GC-1/ANKRD49-Flag cells while the levels of p62 are increased. We also introduced two siRNAs that target different regions of the NF-κB/p65 transcript in GC-1/ANKRD49-Flag cells to attenuate the expression of endogenous NF-κB/p65. Western blot analysis shows that p65 expression is effectively down-regulated (Fig 6C). Similarly, Western blot analysis reveals reduction of Beclin 1, LC3-Ⅱ and increase of p62, along with a decrease in the levels of p65 (Fig 6C). Furthermore, GC-1/ANKRD49-Flag and parental cells transfected with GFP-LC3 were also treated by serum starvation with or without PDTC and BAY 11–7082. As shown in Fig 6D, marked punctate accumulation of GFP-LC3 is observed in GC-1/ANKRD49-Flag cells, while the punctated GFP-LC3 is obviously decreased by PDTC or BAY 11–7082 treatment, showing inhibition of NF-κB pathway in the ANKRD49-induced GC-1 cells directly reduced autophagy.

**Fig 7:**
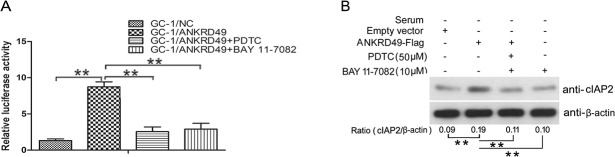
ANKRD49 enhanced the transcriptional activity of NF-κB in serum-starved GC-1 cells. (A) GC-1/Con and GC-1/ANKRD49-Flag stable cells were transfected with an NF-κB–driven luciferase reporter and then cultured in serum-free medium with or without PDTC and BAY 11–7082 for 24 h. NF-κB activation was detected by luciferase reporter assay. Data are compared between indicated groups. n = 3; **p < 0.01. (B) GC-1/Con and GC-1/ANKRD49-Flag stable cells were cultured in serum-free medium with or without PDTC and BAY 11–7082 for 24 h. The levels of cIAP2 were determined by Western blot. β-actin served as a loading control. One representative of three independent experiments is shown. The quantitative results are presented as the ratio of cIAP2 to β-actin (n = 3). **p < 0.01 indicates significant difference between groups as shown.

In addition, we performed dual luciferase reporter assays to assess whether ANKRD49 can enhance the transcriptional activation of NF-κB under serum starvation and whether PDTC or BAY 11–7082 can reverse it. As shown in Fig 7A, ANKRD49 caused an approximate 8-fold increase in relative luciferase activity of NF-κB reporter, and PDTC or BAY 11–7082 greatly inhibited ANKRD49-induced NF-κB transcriptional activity. To further verify the effect of ANKRD49 on NF-κB activity, we measured the levels of cIAP2 which is a NF-κB target gene in response to cellular starvation [27, 28]. As shown in Fig 7B, ANKRD49 increased the levels of cIAP2 in GC-1 cells under serum starvation, and PDTC or BAY 11–7082 attenuated the role of ANKRD49. Taken together, these results show that autophagy regulated by ANKRD49 and induced by serum starvation in GC-1 cells is dependent on the NF-κB pathway.

## Discussion

Mammalian spermatogenesis is a paradigm for the process of development. Genetic information from male germ stem cells is edited, organized and distributed into spermatozoa in a strictly regulated system of sophisticated and well-coordinated gene expressions [29, 30]. The most important functions in spermatogenesis are performed by numerous genes that are located in the testes or germ cells [31]. Thus, identifying and characterising specific genes in the testes will help elucidate the mechanism of spermatogenesis.

In present study, we have identified ANKRD49 as a protein that is highly expressed in mouse testes by showing the expression pattern of ANKRD49. Our finding demonstrates that ANKRD49 is more abundant in adult mouse testes compared to other tissues. It appears at the beginning of testes development. Furthermore, we have examined the distribution of ANKRD49 in the reproductive system and have found that ANKRD49 is predominantly located in the nuclei of spermatogonia, spermatocytes and round spermatids. These results indicate that ANKRD49 may function as a modulator in processes required for spermatogenesis, including cell proliferation, differentiation, apoptosis and autophagy.

It is well established that programmed cell death (PCD) plays a principal role in processes of mammalian spermatogenesis [32]. Apoptosis, a type of PCD, plays a primary role during the different stages of spermatogenesis and has been widely studied [16, 33, 34]. However, the role of autophagy, another type of PCD which is equally important in spermatogenesis [35], still remains to be explored [36, 37]. Basal autophagy plays a critical role in cellular homeostasis by eliminating excessive proteins and organelles [38]. However, the roles of autophagy in cellular death and survival are complex and context-dependent. Autophagy can serve as a survival mechanism during nutrient deprivation or metabolic stress, whereas it can also lead to cellular death (termed autophagic cell death) [39].

Given that autophagy has an important function in spermatogenesis [8], we have investigated the involvement of ANKRD49 in germ cell autophagy. It is difficult to obtain a sufficient amount of highly purified primary spermatogonia cells for experimental purposes. Therefore, we have examined autophagy in a mouse-derived spermatogonia cell line, GC-1 spg. The GC-1 spg cell is a widely used *in vitro* cell model [40] that has the ability to differentiate into mature spermatids [16]. Our findings demonstrate that ANKRD49 participates in serum starvation-induced autophagy of GC-1 cells. It appears that ANKRD49 enhances autophagy that is induced by nutrient deprivation, for GC-1 cells expressing ANKRD49 are more sensitive to nutrient deprivation-induced autophagy while GC-1 cells expressing ANKRD49 shRNA are more resistant.

The NF-κB pathway is involved in control of inflammation, stress response and other physiological processes in cellular signalling. It has a dual role in regulating autophagy. It can serve as both positive [41, 42] and negative regulator of autophagy [24, 43]. We have further examined the association of NF-κB and autophagy in GC-1 cells. Decreased expression of Beclin 1 and LC3-Ⅱ is observed in GC-1/ANKRD49-Flag cells where NF-κB signalling is inhibited by PDTC, BAY11-7082 or siRNA-mediated knockdown of RELA/p65. Moreover, luciferase reporter assay also shows that the transcriptional activity of NF-κB is activated by serum starvation and overexpression of ANKRD49, while PDTC and BAY 11–7082 can inhibit NF-κB’s activity and its target gene cIAP2 expression. These findings indicate that ANKRD49-regulated autophagy of GC-1 cells is dependent on the NF-κB pathway.

The role for autophagy in cell survival or death remains contraversial. In our study, we found that ANKRD49 has a cytoprotective role in nutrient-deprived GC-1 cells by inducing autophagy through the NF-κB pathway. Further investigations are still needed to define the relationship between ANKRD49 and other known signalling pathways involved in spermatogenesis.
